# Acute Flaccid Myelitis Surveillance — United States, January 2020–December 2025

**DOI:** 10.15585/mmwr.mm7526a1

**Published:** 2026-07-09

**Authors:** Adriana S. Lopez, Randall English, Shannon L. Rogers, Brian Emery, Leah A. Goldstein, Claire M. Midgley, Heidi L. Moline, Terry Fei Fan Ng, New Vaccine Surveillance Network Collaborators, New Vaccine Surveillance Network Collaborators, Leila C. Sahni, Julie A. Boom, Natasha B. Halasa, Laura S. Stewart, Eileen J. Klein, Janet A. Englund, Geoffrey A. Weinberg, Peter G. Szilagyi, Rangaraj Selvarangan, Jennifer E. Schuster, John V. Williams, Marian G. Michaels, Mary A. Staat, Daniel Payne

**Affiliations:** 1 National Center for Immunization and Respiratory Diseases, CDC.; Baylor College of Medicine and Texas Children’s Hospital, Houston, Texas; Baylor College of Medicine, Houston, Texas; Vanderbilt University Medical Center, Nashville, Tennessee; Vanderbilt University Medical Center, Nashville, Tennessee; Seattle Children’s Hospital, Seattle, Washington; Seattle Children’s Hospital, Seattle, Washington; University of Rochester School of Medicine and Dentistry, Rochester, New York; University of Rochester School of Medicine and Dentistry, Rochester, New York and University of California at Los Angeles, Los Angeles, California; Children’s Mercy Kansas City, Kansas City, Missouri; Children’s Mercy Kansas City, Kansas City, Missouri; UPMC Children’s Hospital of Pittsburgh, Pittsburgh, Pennsylvania; UPMC Children’s Hospital of Pittsburgh, Pittsburgh, Pennsylvania; Cincinnati Children’s Hospital Medical Center, Cincinnati, Ohio; Cincinnati Children’s Hospital Medical Center, Cincinnati, Ohio

**Summary**

**What is already known about this topic?**

Acute flaccid myelitis (AFM) is a rare but serious neurologic condition that has been associated with enterovirus D68 (EV-D68). EV-D68 circulation was associated with U.S. peaks in AFM cases in 2014, 2016, and 2018 (120–238 cases per year). AFM is clinically and radiologically indistinguishable from paralytic poliomyelitis caused by poliovirus.

**What is added by this report?**

During 2020–2025, 17–48 AFM cases were reported annually, despite increases in EV-D68–associated respiratory illnesses in 2022, 2024, and 2025. Approximately one half of AFM patients had stool specimens tested for poliovirus; 75%–100% of AFM patients had received ≥3 polio vaccine doses. One polio case was identified in New York in 2022.

**What are the implications for public health practice?**

Remaining current with polio vaccinations can reduce the risk for poliovirus infection. To ensure detection of polio cases, stool specimens should be collected and tested from all patients suspected of having AFM. Reporting AFM cases to health departments is important for AFM and polio surveillance. Continued AFM surveillance is needed to further understand the epidemiology of AFM and its association with EV-D68.

## Abstract

Acute flaccid myelitis (AFM) is a rare but serious neurologic condition that causes paralysis and primarily affects children. Clinically and radiologically, AFM is indistinguishable from the acute flaccid paralysis of poliomyelitis caused by poliovirus. Nationwide surveillance for AFM has been conducted in the United States since 2014, when an increase in AFM was first recognized. Laboratory testing and epidemiologic and clinical data collected through surveillance suggest that enteroviruses, particularly enterovirus D68 (EV-D68), are a common cause of AFM. EV-D68 circulation was associated with biennial peaks in AFM cases in the United States in 2014, 2016, and 2018. This report provides an update to nationwide surveillance of confirmed AFM cases during January 2020–December 2025. During this period, the number of confirmed AFM cases reported to CDC remained low (17–48 cases per year) compared with 2014, 2016, and 2018 (120–238 cases per year). Despite ongoing seasonal enterovirus circulation, including increases in EV-D68–associated respiratory illnesses identified from sentinel surveillance sites in 2022, 2024, and 2025, concurrent increases in AFM cases in nationwide surveillance data were not observed; EV-D68 was detected in one AFM patient specimen received by CDC during this period. A majority (approximately 75%) of confirmed cases of AFM occurred in persons who were reported to be up to date with polio vaccination; approximately one half had stool specimens collected and tested. In 2022, a polio case in an unvaccinated person was identified by testing stool specimens collected through AFM surveillance. Clinicians should ensure that stool samples are collected from patients with acute onset of flaccid weakness or paralysis and report all cases to their local health department.

## Introduction

### Acute Flaccid Paralysis

Acute flaccid paralysis (AFP) is a clinical syndrome characterized by sudden onset of weakness and reduced muscle tone. AFP is a generalized term for clinical conditions with both noninfectious etiologies (e.g., transverse myelitis and Guillain Barré syndrome) and infectious etiologies (e.g., poliovirus, enteroviruses, and West Nile virus). Surveillance for AFP is not routinely conducted in the United States because poliomyelitis caused by wild poliovirus has been eliminated in the country (*1*).

### Acute Flaccid Myelitis

Acute flaccid myelitis (AFM) is a subtype of AFP characterized by acute onset of flaccid weakness in one or more limbs and abnormalities in the spinal cord gray matter. Surveillance for AFM has been conducted in the United States since 2014 (*2*). Although many pathogens can cause AFM, laboratory testing and epidemiologic and clinical data collected through surveillance suggest that enteroviruses, particularly enterovirus D68 (EV-D68), are a common cause. Biennial peaks in AFM cases in the United States in 2014, 2016, and 2018 were associated with EV-D68 circulation (*2*). Because AFM is clinically and radiologically indistinguishable from polio, cases that meet criteria for AFM detected through AFM surveillance in the United States are also assessed to rule out poliomyelitis caused by poliovirus. Continued AFM surveillance is needed to further understand the epidemiology of AFM and to ensure that the United States remains polio-free. This report provides a surveillance update on confirmed AFM cases reported to CDC during January 2020–December 2025.

## Methods

### Data Sources

**Clinical and radiologic data.** As part of nationwide AFM surveillance, U.S. jurisdictions report cases that meet criteria for AFM to CDC. These criteria include 1) acute onset of flaccid limb weakness and 2) the presence of any spinal cord gray matter lesions detected by magnetic resonance imaging (MRI). Health department personnel complete a patient summary form with demographic and clinical information and gather selected elements from patients’ medical records for data abstraction at CDC. For surveillance purposes, a confirmed case of AFM is defined as the acute onset of flaccid limb weakness in a patient who received an MRI result indicating a spinal cord lesion largely restricted to gray matter and spanning one or more vertebral segments (*3*).

**Vaccination data.** To assess the risk for poliomyelitis in a patient with AFM, polio vaccination status is collected using data from health departments, vaccination registries, or patient medical records. Patients who have not received any doses of polio vaccine are considered to be at highest risk for polio.

**Laboratory data.** When available, cerebrospinal fluid (CSF), respiratory, serum, and stool specimens are submitted to CDC for testing for enteroviruses (including poliovirus) and rhinoviruses using previously described methods (*4*). Enterovirus and rhinovirus test results include those from CDC or external laboratories. Stool specimens are also tested for poliovirus by virus isolation in cell culture. If a stool specimen from a patient with AFM tests positive for poliovirus, that patient is classified as having a confirmed case of paralytic poliomyelitis and not AFM. Since 2017, surveillance for EV-D68 detections in respiratory specimens has been actively conducted through systematic testing provided for children with acute respiratory illness in the New Vaccine Surveillance Network (NVSN) (*5*).

### Data Analysis

Analyses of demographic, clinical, and laboratory data were conducted to describe characteristics of U.S. patients with AFM during 2020–2025. Up-to-date polio vaccination status was defined as receipt of ≥3 polio vaccine doses, verified through vaccination registry data or medical record data. Data from NVSN were reviewed to monitor circulation of EV-D68 and detect possible associations with increases in AFM cases. This activity was reviewed by CDC, deemed not research, and conducted consistent with applicable federal law and CDC policy.*

## Results

### Reported AFM Cases

During 2020–2025, a total of 172 confirmed AFM cases were reported to CDC, including 34 in 2020, 29 in 2021, 48 in 2022, 19 in 2023, 25 in 2024, and 17 in 2025 (Figure) (Table 1). Annually, approximately 75% of patients with confirmed AFM during 2020–2025 were aged <18 years. In that group, the median age was 8 years in 2020, 2021, and 2024; 7 years in 2022; 12 years in 2023; and 9 years in 2025.

**FIGURE:**
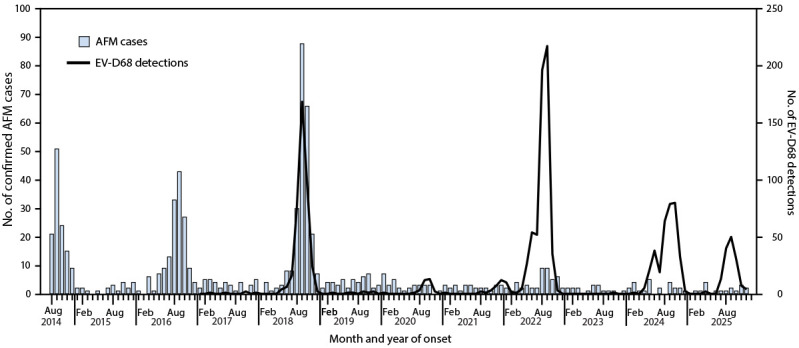
Number of confirmed cases of acute flaccid myelitis from nationwide surveillance,* August 2014–December 2025, and enterovirus D68 detections in respiratory specimens — New Vaccine Surveillance Network,^†^ United States, January 2017–December 2025^§^ **Abbreviations:** AFM = acute flaccid myelitis; EV-D68 = enterovirus D68. * AFM surveillance began in August 2014. ^†^ Testing data from children and adolescents aged <18 years evaluated at an enrollment facility (outpatient clinic, emergency department, or inpatient) at one of seven U.S. pediatric medical centers (Seattle, Washington; Kansas City, Missouri; Nashville, Tennessee; Rochester, New York; Pittsburgh, Pennsylvania; Cincinnati, Ohio; and Houston, Texas) for acute respiratory illness. Testing for EV-D68 began in 2017, was seasonal (July through October or November) at most sites before July 2021, and has been conducted year-round at all sites since July 2021. Two sites (Houston, Texas and Rochester, New York) have had year-round testing for EV-D68 since 2017. Within these testing periods, four of seven sites only performed EV-D68 testing for specimens that were positive for rhinovirus, enterovirus, or both, whereas the remaining sites tested all acute respiratory illness specimens for EV-D68 directly. ^§^ Surveillance for enterovirus D68 began in January 2017.

**TABLE 1:** Number and percentage of patients with confirmed acute flaccid myelitis, by selected demographic and clinical characteristics and year — United States, 2020–2025

Characteristic	2020 N = 34	2021 N = 29	2022 N = 48	2023 N = 19	2024 N = 25	2025 N = 17
**Age group, yrs**						
<18, no. (%)	31 (91)	24 (83)	39 (81)	16 (84)	23 (92)	13 (76)
Median age of patients <18 (IQR)	8 (3–11)	8 (5–13)	7 (5–12)	12 (9–15)	8 (2–12)	9 (3–14)
Median age of all patients (IQR)	9 (3–14)	9 (5–16)	11 (6–14)	13 (10–18)	9 (2–13)	11 (6–18)
**Sex, no. (%)**						
Female	17 (50)	17 (59)	21 (44)	8 (42)	15 (60)	10 (59)
Male	17 (50)	12 (41)	27 (56)	10 (53)	10 (40)	7 (41)
**Race and ethnicity, no. (%)**						
American Indian or Alaska Native	0 (—)	0 (—)	0 (—)	0 (—)	0 (—)	0 (—)
Asian	3 (9)	0 (—)	3 (6)	2 (11)	1 (4)	0 (—)
Black or African American	4 (12)	2 (7)	5 (10)	1 (5)	4 (16)	2 (12)
Hispanic or Latino	9 (26)	10 (34)	6 (12)	7 (37)	7 (28)	2 (12)
Native Hawaiian or Pacific Islander	0 (—)	0 (—)	0 (—)	0 (—)	0 (—)	0 (—)
White	13 (38)	12 (41)	28 (58)	8 (42)	13 (52)	12 (71)
Multiple races	0 (—)	1 (3)	1 (2)	0 (—)	0 (—)	0 (—)
Unknown	5 (15)	4 (14)	5 (10)	1 (5)	0 (—)	1 (6)
**U.S. Census Bureau region, no. (%)**						
Northeast	6 (18)	8 (28)	10 (21)	1 (5)	9 (36)	3 (18)
Midwest	7 (21)	4 (14)	8 (17)	4 (21)	2 (8)	3 (18)
South	12 (35)	11 (38)	14 (29)	7 (37)	10 (40)	6 (35)
West	9 (26)	6 (21)	16 (33)	7 (37)	4 (16)	5 (29)
**Limbs affected, no. (%)**						
Upper	20 (59)	19 (66)	36 (75)	8 (42)	11 (44)	8 (47)
Lower	28 (82)	26 (90)	31 (65)	19 (100)	21 (84)	16 (94)
**Illness during 4 weeks before limb weakness onset, no. (%)**						
Any illness	21 (62)	21 (72)	40 (83)	11 (58)	19 (76)	14 (82)
Respiratory illness	15 (44)	14 (48)	29 (60)	7 (37)	15 (60)	11 (65)
Fever	13 (38)	9 (31)	23 (48)	4 (21)	11 (44)	9 (53)
Respiratory illness or fever	21 (62)	17 (59)	38 (79)	8 (42)	18 (72)	13 (76)
Gastrointestinal illness	3 (9)	10 (34)	13 (27)	6 (32)	4 (16)	6 (35)
**Median days from prior illness to limb weakness, no. (IQR)**						
Any illness	6 (2–12)	6 (3–10)	6 (3–8)	12 (2–20)	7 (2–12)	3 (1–7)
Respiratory illness	6 (2–14)	5 (3–8)	6 (4–8)	18 (2–20)	8 (2–11)	3 (1–7)
Fever	2 (1–6)	3 (2–15)	3 (1–8)	3 (1–15)	4 (1–10)	1 (0–4)
Respiratory illness or fever	6 (2–12)	5 (3–8)	6 (3–8)	10 (1–19)	8 (2–12)	3 (0–6)
Gastrointestinal illness	4 (0–14)	4 (0–7)	2 (0–9)	6 (3–20)	3 (0–6)	0 (0–6)
**Cerebrospinal fluid microscopic examination, no. (%) **	28 (100)	27 (100)	42 (100)	18 (100)	18 (100)	14 (100)
Pleocytosis, no. (%)	14 (50)	12 (44)	29 (69)	9 (50)	13 (72)	9 (64)
Median white blood cells per mm^3^ (IQR)	28 (9–64)	37 (16–86)	53 (24–129)	155 (107–365)	91 (46–156)	94 (27-103)
**Polio vaccination, no. (%)**						
Status						
IPV or OPV	21 (62)	24 (83)	43 (90)	19 (100)	21 (84)	11 (65)
Unvaccinated	0 (—)	0 (—)	1 (2)	0 (—)	2 (8)	1 (6)
Unknown vaccination status	13 (38)	5 (17)	4 (8)	0 (—)	2 (8)	5 (29)
Doses received (among those vaccinated)						
≥3 doses (up to date)	20 (95)	18 (75)	38 (88)	16 (84)	17 (81)	11 (100)
1–2 doses	1 (5)	4 (17)	0 (—)	1 (5)	1 (5)	0 (—)
Unknown	0 (—)	2 (8)	5 (12)	2 (11)	3 (14)	0 (—)
**Hospitalizations and treatment**						
Hospitalizations, no. (%)	34 (100)	29 (100)	47 (98)	19 (100)	25 (100)	17 (100)
Hospitalization before or after limb weakness onset, no. (%)						
Before hospitalization	1 (3)	0 (—)	3 (6)	1 (5)	1 (4)	0 (—)
After hospitalization	33 (97)	29 (100)	44 (94)	18 (95)	24 (96)	17 (100)
Unknown	0 (—)	0 (—)	0 (—)	0 (—)	0 (—)	0 (—)
Days from limb weakness onset to hospitalization (among those hospitalized after onset)						
Hospitalized after onset, no. (%)	33 (100)	29 (100)	44 (100)	18 (100)	24 (100)	17 (100)
Median (IQR)	1 (1–1)	1 (0–1)	1 (1–3)	1 (0–1)	1 (0–3)	1 (0–2)
0–1 days, no. (%)	28 (85)	24 (83)	27 (61)	15 (83)	14 (58)	13 (76)
2–3 days, no. (%)	4 (12)	3 (10)	11 (25)	2 (11)	7 (29)	3 (18)
4–7 days, no. (%)	0 (—)	1 (3)	4 (9)	1 (6)	2 (8)	0 (—)
>7 days, no. (%)	1 (3)	1 (3)	2 (5)	0 (—)	1 (4)	1 (6)
Treatment, no. (%)						
Steroids, no IVIG	8 (24)	2 (7)	11 (23)	3 (16)	4 (16)	3 (18)
IVIG, no steroids	6 (18)	8 (28)	10 (21)	1 (5)	3 (12)	3 (18)
Steroids and IVIG	14 (41)	17 (59)	25 (52)	11 (58)	13 (52)	10 (59)
Plasma exchange	11 (32)	7 (24)	12 (25)	8 (42)	14 (56)	11 (65)
Admitted to ICU	20 (59)	21 (72)	24 (50)	9 (47)	16 (64)	13 (76)
Respiratory support	6 (18)	8 (28)	11 (23)	5 (26)	5 (20)	8 (47)
Mechanical ventilation	5 (15)	7 (24)	9 (19)	3 (16)	4 (16)	7 (41)
**First health care visit after limb weakness onset **						
Location, no. (%)						
Emergency department	24 (71)	19 (66)	30 (62)	12 (63)	17 (68)	16 (94)
Primary care provider	4 (12)	2 (7)	4 (8)	2 (11)	2 (8)	0 (—)
Urgent care provider	1 (3)	3 (10)	2 (4)	1 (5)	2 (8)	1 (6)
Weakness onset during hospitalization	1 (3)	0 (—)	3 (6)	1 (5)	1 (4)	0 (—)
Unknown or other	4 (12)	5 (17)	9 (19)	3 (16)	3 (12)	0 (—)
Days from onset to first health care visit (excludes hospitalizations before onset)						
First visit after onset, no. (%)	33 (100)	29 (100)	45 (100)	18 (100)	24 (100)	17 (100)
Median (IQR)	0 (0–0)	0 (0–1)	0 (0–1)	0 (0–0)	0 (0–1)	0 (0–1)
0–1 days, no. (%)	32 (97)	26 (90)	33 (73)	17 (94)	17 (71)	16 (94)
2–3 days, no. (%)	0 (—)	2 (7)	6 (13)	0 (—)	4 (17)	0 (—)
4–7 days, no. (%)	0 (—)	1 (3)	2 (4)	0 (—)	1 (4)	0 (—)
>7 days, no. (%)	0 (—)	0 (—)	0 (—)	0 (—)	0 (—)	1 (6)
Unknown, no. (%)	1 (3)	0 (—)	4 (9)	1 (6)	2 (8)	0 (—)

**Abbreviations:** ICU = intensive care unit; IPV = inactivated polio vaccine; IVIG = intravenous immunoglobulin; OPV = oral polio vaccine.

### Clinical Characteristics of Patients with AFM

**Respiratory illness history and CSF findings.** A higher percentage of patients with confirmed AFM in 2022, 2024, and 2025 had history of any respiratory illness or fever (72%–79%) than did patients in 2020, 2021, and 2023 (42%–62%) (Table 1). A higher percentage of patients with confirmed AFM in 2022 and 2024 had CSF pleocytosis (69%–72%) than did patients in 2020, 2021, 2023, and 2025 (44%–64%).

**Limb involvement.** Among patients with AFM in 2022, 75% had any upper limb involvement, and 65% had any lower limb involvement (Table 1). In all other years, lower limb involvement was more common than upper limb involvement.

### Polio Vaccination Status of Patients with AFM

Among patients with confirmed AFM and documented receipt of any polio vaccine doses, up-to-date polio vaccination (receipt of ≥3 vaccine doses) ranged from 75% in 2021 to 100% in 2025. The percentage of patients who had received 1–2 doses of polio vaccine ranged from 0% in 2022 and 2025 to 17% in 2021; 0% (2020 and 2025) to 14% (2024) had received an unknown number of polio vaccine doses (Table 1). The percentage of AFM patients with unknown vaccination status ranged from 0% (2023) to 38% (2020); among these patients, a median of 60% (range = 0%–100%) were aged ≥18 years.

### Clinical Course

Among all patients with confirmed AFM during 2020–2025, 98%–100% were hospitalized, including 58%–85% who were hospitalized within 1 day of onset of limb weakness (Table 1). Most patients (62%–94%) first sought care in a hospital emergency department. The most common treatments, received by 41%–59% of hospitalized patients with confirmed AFM, were steroids and intravenous immunoglobulin. Admission to an intensive care unit ranged from 47% in 2023 to 76% in 2025. Among patients who were hospitalized, 18%–47% required respiratory support and 15%–41% received invasive mechanical ventilation. No patients with confirmed AFM during 2020–2025 died.

### Laboratory Test Results for Patients with AFM

Among the 172 patients with confirmed AFM reported during 2020–2025, 163 (95%) received laboratory test results from at least one clinical specimen, and a range of 26% (2023) to 55% (2024) of patients received a positive enterovirus or rhinovirus test result from at least one tested specimen (Table 2). Enterovirus or rhinovirus detections were most common in respiratory specimens (58; 40% of all cases); the most common viruses detected were rhinoviruses (24% of all cases positive for enterovirus or rhinovirus) and unknown or untyped enteroviruses (72% of all cases positive for enterovirus or rhinovirus). EV-D68 was detected in the respiratory specimen of one patient in 2025. Enterovirus A-71 was detected in the stool of one patient in 2020 and 2021 and two in 2022. Overall, 95 (55%) patients had stool specimens collected and tested; annual percentages ranged from 44% to 54% in all years except 2023 and 2025, when 74% and 76% of patients, respectively, had stool specimens tested. One case of poliomyelitis caused by poliovirus was detected in New York in 2022 (*6*) and was not included in the count of confirmed AFM cases.

**TABLE 2:** Enterovirus and rhinovirus test results among patients with confirmed acute flaccid myelitis, by specimen type and year — United States, 2020–2025

Specimen type and test results	Year, no. (%)					
	2020 N = 34	2021 N = 29	2022 N = 48	2023 N = 19	2024 N = 25	2025 N = 17
**Total patients with enterovirus and rhinovirus test results**	**32 (94)**	**28 (97)**	**45 (94)**	**19 (100)**	**22 (88)**	**17 (100)**
Patients with any positive result	9 (28)	12 (43)	21 (47)	5 (26)	12 (55)	8 (47)
Enterovirus-D68	0 (—)	0 (—)	0 (—)	0 (—)	0 (—)	1 (12)
Enterovirus-A71	1 (11)	1 (8)	2 (10)	0 (—)	0 (—)	0 (—)
Rhinoviruses	3 (33)	3 (25)	5 (24)	0 (—)	3 (25)	0 (—)
Other typed enteroviruses	0 (—)	2 (17)	3 (14)	0 (—)	0 (—)	0 (—)
Unknown or not typed	5 (56)	6 (50)	11 (52)	5 (100)	9 (75)	7 (88)
**Respiratory specimen**						
Patients with results	29 (85)	27 (93)	38 (79)	17 (89)	19 (76)	15 (88)
Patients with positive results	8 (28)	9 (33)	19 (50)	5 (29)	10 (53)	7 (47)
Enterovirus-D68	0 (—)	0 (—)	0 (—)	0 (—)	0 (—)	1 (14)
Enterovirus-A71	0 (—)	0 (—)	0 (—)	0 (—)	0 (—)	0 (—)
Rhinoviruses	3 (38)	3 (33)	5 (26)	0 (—)	3 (30)	0 (—)
Other typed enteroviruses	0 (—)	0 (—)	1 (5)	0 (—)	0 (—)	0 (—)
Unknown or not typed	5 (62)	6 (67)	13 (68)	5 (100)	7 (70)	6 (86)
**Stool specimen**						
Patients with results	15 (44)	14 (48)	26 (54)	14 (74)	13 (52)	13 (76)
Patients with positive results	2 (13)	3 (21)	5 (19)	0 (—)	3 (23)	1 (8)
Enterovirus-D68	0 (—)	0 (—)	0 (—)	0 (—)	0 (—)	0 (—)
Enterovirus-A71	1 (50)	1 (33)	2 (40)	0 (—)	0 (—)	0 (—)
Rhinoviruses	0 (—)	0 (—)	0 (—)	0 (—)	1 (33)	0 (—)
Other typed enteroviruses	0 (—)	2 (67)	3 (60)	0 (—)	0 (—)	0 (—)
Unknown or not typed	1 (50)	0 (—)	0 (—)	0 (—)	2 (67)	1 (100)
**Cerebrospinal fluid**						
Patients with results	30 (88)	27 (93)	38 (79)	18 (95)	17 (68)	16 (94)
Patients with positive results	0 (—)	1 (4)	0 (—)	0 (—)	0 (—)	1 (6)
Enterovirus-D68	0 (—)	0 (—)	0 (—)	0 (—)	0 (—)	0 (—)
Enterovirus-A71	0 (—)	0 (—)	0 (—)	0 (—)	0 (—)	0 (—)
Rhinoviruses	0 (—)	0 (—)	0 (—)	0 (—)	0 (—)	0 (—)
Other typed enteroviruses	0 (—)	0 (—)	0 (—)	0 (—)	0 (—)	0 (—)
Unknown or not typed	0 (—)	1 (100)	0 (—)	0 (—)	0 (—)	1 (100)
**Serum specimen**						
Patients with results	23 (68)	21 (72)	25 (52)	9 (47)	6 (24)	7 (41)
Patients with positive results	1 (4)	2 (10)	0 (—)	0 (—)	0 (—)	1 (14)
Enterovirus-D68	0 (—)	0 (—)	0 (—)	0 (—)	0 (—)	0 (—)
Enterovirus-A71	1 (100)	0 (—)	0 (—)	0 (—)	0 (—)	0 (—)
Rhinoviruses	0 (—)	0 (—)	0 (—)	0 (—)	0 (—)	0 (—)
Other typed enteroviruses	0 (—)	1 (50)	0 (—)	0 (—)	0 (—)	0 (—)
Unknown or not typed	0 (—)	1 (50)	0 (—)	0 (—)	0 (—)	1 (100)

### Detection of EV-D68 in Children with Acute Respiratory Illnesses

Data from NVSN identified increases in detections of EV-D68 in respiratory specimens from children with acute respiratory illnesses in 2022, 2024, and 2025; however, these detections followed different annual trends from those of AFM cases nationally and were not associated with a concurrent increase in AFM cases (Figure). In addition, EV-D68 was detected in a specimen from one patient with AFM in 2025 (Table 2). NVSN 2025 data indicated that the increase in EV-D68 detections among children with acute respiratory illnesses began in July, reaching 9% of weekly respiratory cases in September 2025.

## Discussion

During January 2020–December 2025, the number of confirmed AFM cases reported to CDC remained low compared with previous years, despite increases of EV-D68–associated respiratory illness in 2022, 2024, and 2025. Although clinical characteristics of patients with AFM in 2022 and 2024 were similar to those from 2018 (*2*), a year when the large increase in cases was attributed to EV-D68, only one AFM patient during 2020–2025 had EV-D68 detected in any specimens. Although additional patients with AFM during this period might have had EV-D68 infection, detection was limited by timing of specimen collection. Specimens from patients with AFM are typically collected after onset of limb weakness rather than during the prodromal illness that can occur up to 7 days before weakness onset, when viral detection might be more likely. Furthermore, many specimens that tested positive for enterovirus or rhinovirus might not have been typed if they were not submitted to CDC for sequencing.

During 2020–2021, nonpharmaceutical interventions implemented during the COVID-19 pandemic likely led to decreased circulation of respiratory viruses, including EV-D68 (*7*). Detections of EV-D68 respiratory cases, as measured in NVSN sentinel surveillance sites, returned in 2022, exceeding 2018 levels (*8*), and also increased in 2024 and 2025. NVSN data suggest a possible return to the biennial pattern for EV-D68 circulation (*5*,*9*), whereas AFM cases have remained at baseline levels since 2018. Reasons for this finding remain unclear. One hypothesis is that the EV-D68 strains circulating in 2022, 2024 and 2025 (and possibly in years to come) are less neurovirulent. A recent study found that the predominant EV-D68 strains being detected lack some of the amino acid substitutions in the viral capsid protein VP1 that induce neurovirulence in mouse and neuron models (*9*,*10*). Additional studies that directly test the phenotype of recent amino acid changes are warranted to assess this hypothesis.

Based on a presumed biennial pattern in EV-D68 circulation, the increase in EV-D68 detections beginning in July 2025 was unexpected. Efforts are underway to better understand EV-D68 trends both locally and across the NVSN sites after possible pandemic interruptions.

Because AFM and poliomyelitis caused by poliovirus are clinically and radiologically indistinguishable, collecting stool specimens from patients who meet the criteria for AFM is critical to rule out polio. During 2020–2025, approximately one half of patients with confirmed AFM cases had stool specimens collected and tested for poliovirus, which is less than the 80% recommended for AFP surveillance internationally. However, in the United States, the health care system provides access to resources (e.g., imaging and laboratory testing) that providers can use to develop alternative diagnoses for cases of AFP. Furthermore, 75%–100% of patients with confirmed AFM were up to date with polio vaccination and considered to be at low risk for having polio.

In 2022, a case of paralytic poliomyelitis was identified in a person in New York (*6*). The patient was an unvaccinated young adult who was initially evaluated as having a possible case of AFM. Following AFM surveillance protocol, serum, CSF, and respiratory and stool specimens were collected for testing and confirmed that the patient had poliomyelitis caused by poliovirus. Because the patient had no history of international travel, and the last case of indigenous polio in the United States occurred in 1979, poliomyelitis caused by poliovirus was not initially suspected. Poliovirus circulates in the environment for months after being shed in the stool of an infected person and can infect susceptible persons. Polio vaccination is critical for decreasing the number of cases in the community and preventing paralytic polio. The occurrence of this case underscores the importance of collection of stool specimens for testing from all patients with suspected AFM, especially from persons who are not up to date with their polio vaccinations; however, only approximately one half of patients with confirmed AFM had stool collected and tested in recent years, with the exception of 2023 (74%), possibly because of heightened awareness after the occurrence of the 2022 polio case, and 2025 (76%). Few patients with AFM were completely unvaccinated (2%) or undervaccinated (5%). Because patients whose vaccination status was unknown were more likely to be aged ≥18 years, they might have been immune to polio but did not have vaccination documentation of or could not remember being vaccinated.

### Limitations

The findings in this report are subject to at least three limitations. First, the clinical data for this analysis were collected through a patient summary form completed by health department personnel and through a review of the patient’s clinical records; therefore, information might be incomplete. Second, although efforts were made to validate polio vaccination statuses through vaccination registries, data were not always available. These unknown records might have resulted in an underestimate of the percentage of persons who were vaccinated. Finally, cases with suspected AFM are passively reported to CDC, which could result in an underestimate of the number of AFM cases in the United States.

### Implications for Public Health Practice

The timing of the next increase in AFM remains uncertain, especially because the 2022, 2024, and 2025 increases in respiratory detections of EV-D68 were not associated with a concurrent increase in AFM. Given the unexpected EV-D68 increase in 2025, clinicians should remain alert for patients suspected of having AFM and report these cases to their local or state health department. In addition, persons should remain up to date with polio vaccination to reduce their risk for infection with poliovirus. Because AFM and poliomyelitis caused by poliovirus are clinically and radiologically indistinguishable, obtaining polio vaccination status, collecting stool specimens from patients suspected of having AFM, and retaining high polio vaccination levels are critical for detecting and maintaining the elimination of polio in the United States.

## Footnotes

* 45 C.F.R. part 46.102(l)(2), 21 C.F.R. part 56; 42 U.S.C. Sect. 241(d); 5 U.S.C. Sect. 552a; 44 U.S.C. Sect. 3501 et seq.
